# Potential for Nuclear Medicine Therapy for Glioblastoma Treatment

**DOI:** 10.3389/fphar.2019.00772

**Published:** 2019-07-10

**Authors:** Clément Bailly, Aurelien Vidal, Coralie Bonnemaire, Françoise Kraeber-Bodéré, Michel Chérel, Amandine Pallardy, Caroline Rousseau, Emmanuel Garcion, Franck Lacoeuille, François Hindré, Samuel Valable, Myriam Bernaudin, Caroline Bodet-Milin, Mickaël Bourgeois

**Affiliations:** ^1^Nuclear Medicine, Centre Hospitalier Universitaire (CHU) de Nantes, Nantes, France; ^2^CRCINA, INSERM, CNRS, Université d'Angers, Université de Nantes, Nantes, France; ^3^Arronax, Saint-Herblain, France; ^4^Nuclear Medecine, Centre Hospitalier Universitaire (CHU) de Nantes, Nantes, France; ^5^Institut de Cancérologie de l’Ouest (ICO), Angers, France; ^6^Team 17—Design and Application of Innovative Local Treatments in Glioblastoma, INSERM U1232 Centre de Recherche en Cancérologie et Immunologie Nantes Angers (CRCINA), Nantes, France; ^7^Nuclear Medicine, Centre Hospitalier Universitaire d’Angers, Angers, France; ^8^Normandie Université, Caen, France

**Keywords:** glioblastoma, nuclear medicine, cancer, radioimmunotherapy (RIT), peptide receptor radiotherapy (PRRT), radionanoparticles

## Abstract

Glioblastoma is the most common malignant adult brain tumor and has a very poor patient prognosis. The mean survival for highly proliferative glioblastoma is only 10 to 14 months despite an aggressive current therapeutic approach known as Stupp’s protocol, which consists of debulking surgery followed by radiotherapy and chemotherapy. Despite several clinical trials using anti-angiogenic targeted therapies, glioblastoma medical care remains without major progress in the last decade. Recent progress in nuclear medicine, has been mainly driven by advances in biotechnologies such as radioimmunotherapy, radiopeptide therapy, and radionanoparticles, and these bring a new promising arsenal for glioblastoma therapy. For therapeutic purposes, nuclear medicine practitioners classically use β^−^ particle emitters like ^131^I, ^90^Y, ^186/188^Re, or ^177^Lu. In the glioblastoma field, these radioisotopes are coupled with nanoparticles, monoclonal antibodies, or peptides. These radiopharmaceutical compounds have resulted in a stabilization and/or improvement of the neurological status with only transient side effects. In nuclear medicine, the glioblastoma-localized and targeted internal radiotherapy proof-of-concept stage has been successfully demonstrated using β^−^ emitting isotopes. Similarly, α particle emitters like ^213^Bi, ^211^At, or ^225^Ac appear to be an innovative and interesting alternative. Indeed, α particles deliver a high proportion of their energy inside or at close proximity to the targeted cells (within a few micrometers from the emission point versus several millimeters for β^−^ particles). This physical property is based on particle–matter interaction differences and results in α particles being highly efficient in killing tumor cells with minimal irradiation of healthy tissues and permits targeting of isolated tumor cells. The first clinical trials confirmed this idea and showed good therapeutic efficacy and less side effects, thus opening a new and promising era for glioblastoma medical care using α therapy. The objective of this literature review is focused on the developing field of nuclear medicine and aims to describe the various parameters such as targets, vectors, isotopes, or injection route (systemic and local) in relation to the clinical and preclinical results in glioblastoma pathology.

## Introduction

Glioblastoma is a neoplasm derived from astrocytes, a subtype of brain macroglial cells. Historically, astrocytomas from the most benign to the most aggressive tumors have been classified using four grades (Louis et al., 2016): pilocytic astrocytoma (grade I tumors), diffuse astrocytoma (grade II tumors), anaplastic astrocytoma (grade III tumors), and glioblastoma (grade IV tumors). Glioblastoma appears to be the most aggressive and also, unfortunately, the most frequent primary brain tumor. The worldwide incidence of glioblastoma is less than 10 per 100,000 people (Hanif et al., 2017; Tamimi and Juweid, 2017) and represents approximately 50–60% of gliomas and 15% of all primary brain tumors in adults (Ostrom et al., 2016; Tamimi and Juweid, 2017; Li et al., 2018). Currently, glioblastoma can be divided into different subtypes based on molecular classification, which includes an isocitrate dehydrogenase-1 (IDH) mutation described in the 2016 WHO classification (Louis et al., 2016), or the more recently described 1p/19q codeletion and TERT promoter mutation (Eckel-Passow et al., 2015; Karsy et al., 2017; van den Bent et al., 2017). The current standardized therapeutic protocol known as Stupp’s protocol consists of a debulking surgery followed by radiotherapy and chemotherapy (Stupp et al., 2005). Despite the increase in the molecular knowledge of the pathology and the emergence of targeted therapies with some clinical trials based on this molecular stratification (Chen et al., 2017), patient outcomes remain poor with a survival rate of 14–15 months after diagnosis (Thakkar et al., 2014; Kaley et al., 2018), and there has been no significant progress made in the last decade.

The recent progress in nuclear medicine development has generated a new promising arsenal for glioblastoma therapy. This has been mainly driven by biotechnologies such as radioimmunotherapy, radiopeptide therapy, and radionanoparticles. The four main parameters required for successful radionuclide targeted therapies for glioblastoma are the selection of an appropriate target, the size of the targeting vector, the physical properties of the radionuclide, and the physicochemical properties of the vector (Cordier et al., 2016). The objective of this literature review is focused on the improving field of nuclear medicine and describes the various parameters such as targets, vectors, isotopes, or injection route (systemic and local) in relation to clinical and preclinical results in glioblastoma pathology.

## Nuclear Medicine and Radiopharmaceuticals

Nuclear medicine is the medical specialty that uses radioactive atoms for diagnosis and/or therapy. In the therapeutic case, to obtain specific irradiation of tumor cells, the radioactivity could be attached to a pharmaceutical molecule that binds to specific molecules expressed on the target tumor cells. This specific radioactive molecule is called a radiopharmaceutical, and currently, we have many possible combinations. The pharmacological specific component of a radiopharmaceutical in glioblastoma therapy can be based on the target protein structure and includes peptides or monoclonal antibodies, or molecular structures like nanoparticles. For the radioactive side of therapeutic radiopharmaceuticals, nuclear medicine practitioners can use massive particle emitters, which deliver their ionizing energy locally like Auger electrons, or β^−^ or α particles. Auger electrons are low-energy electrons that emit a very localized irradiation (several nanometers around the emission point) with high biological effects. Beta-negative particles have a relatively (compared with alpha particles) low linear energy transfer (LET) and emit their energy over a few millimeters. Radionuclide choice is based on the tumor size. For example, yttrium-90 emits a long-range beta emission and could be used for larger masses, while lutetium-177 has a short range, favoring treatment of minimal residual disease.

Alpha particles deliver a high fraction of their energy inside the targeted cells, leading to highly efficient killing. This makes them suitable for targeting isolated tumor cells and minimal residual disease. The physical properties of radionuclides used for glioblastoma are summarized in **Table 1**.

**Table 1:** Physical properties of radioisotopes used in glioblastoma therapy.

Radionuclide	Emission type	Half-life (h)	*E*_max_ (keV) of main emission	Maximum range in soft tissues (mm)
Iodine-125	Auger	1426	3.19	Nanometer scale
Iodine-131	β^−^	193	606.3	2.9
Yttrium-90	β^−^	64	2,280.1	12.0
Lutetium-177	β^−^	162	498.3	2.0
Rhenium-186	β^−^	89.2	1,069.5	5.0
Rhenium-188	β^−^	17	2,120.4	10.8
Astatine-211	α	7.2	5.870 to 7.45	0.055 to 0.080
Bismuth-213	α	0.76	8.4	0.1
Actinium-225	α	240	8.4	0.1

### Radioimmunotherapy Approach

Radioimmunotherapy (RIT) is a nuclear medicine modality that uses a monoclonal antibody (mAb) to achieve targeted vectorization of a radionuclide. The monoclonal antibody binds to specific antigens expressed or overexpressed on the tumor cells. For certain antigen targets, like epidermal growth factor receptor (EGFR), tenascin, or DNA histone H1 complex, clinical trials are underway; and the initial results show promise. Additional targets such as CA XII or cadherin 5 are also showing encouraging results at the preclinical stage.

#### GFR Targeting

EGFR is a cell-surface receptor involved in regulation of cell proliferation, angiogenesis, and tumor metastases. EGFR is particularly overexpressed in over 90% of glioblastomas and constitutes an interesting target for glioblastoma RIT (Frederick et al., 2000). One of the first glioblastoma RIT clinical trials using a mAb directed against the EGFR antigen and radiolabeled with iodine-125 showed a significant and promising increase in median survival. In this phase II clinical study, 180 patients, of which 118 had a glioblastoma diagnosis, received intravenous or intra-arterial RIT as an adjuvant therapy after surgery, radiotherapy, and with or without chemotherapy. The mean total administered dose was 5.2 GBq (one injection per week for 3 weeks). The overall median survival for the glioblastoma group was 13.4 months, demonstrating a significant outcome improvement. Furthermore, a subgroup of patients less than 40 years old showed a median survival of 25.4 months (Emrich et al., 2002). This first RIT application was confirmed by a second phase II clinical trial, which involved 192 patients with glioblastoma. Patients received 1.8 GBq each week for 3 weeks (total of 4.44 to 5.55 GBq) of RIT after debulking surgery and radiation therapy. The results showed no grade 3/4 toxicological events and an overall median survival of 15.7 months with an increment to 20.2 months for the arm with temozolomide and RIT-associated therapy (Li et al., 2010).

Another EGFR RIT targeting modality was tested by Casacó et al. in 2008 using a single-dose intracavitary injection of anti-EGFR (nimotuzumab) radiolabeled with rhenium-188. This phase I clinical trial included eight patients with glioblastoma multiform. The maximal tolerated dose was determined at 370 MBq for 3 mg of mAb. In the 370 MBq group, two patients presented a complete response after 3 years of monitoring, and one patient presented a partial response for more than 1 year. However, no improvement in median survival was reported due to the large variability (from 6.1 to 18.7 months), and a transient to very severe neurotoxicity was also observed (Casacó et al., 2008).

#### Tenascin Targeting

The extracellular matrix protein tenascin-C is another target of interest for glioblastoma RIT. Tenascin-C is overexpressed in more than 90% of all glioblastoma cases, and this protein is involved in adhesion, migration, and proliferation with increased proliferation with higher grades of tumor malignancy (Zagzag et al., 1996; Midwood et al., 2016).

The iodine-131-radiolabeled anti-tenascin mAb BC2 was the first used in this indication for 10 patients with recurring glioblastoma after surgery, radiotherapy, and chemotherapy. The mean dose of 551.3 MBq for 1.93 mg of mAb was injected directly into the tumor by the stereotaxic method. Both systemic and neurologic toxicities were negligible, and RIT failed to show any favorable results in four patients. Nevertheless, for three patients, the pathology appeared to be stabilized; for two patients, the results revealed a partial remission; and one patient showed complete remission (Riva et al., 1992). This approach was confirmed using other anti-tenascin mAb. BC4 was injected intratumorally in 30 patients with recurrent glioblastoma at a higher dose (1,100 MBq of iodine-131 repeated two, three, or four times) without adverse systemic effects and comparable results (Riva et al., 1994). A major phase I/II clinical trial using iodine-131-labeled mAb BC2 and BC4 enrolled 111 patients who suffered diverse malignant gliomas (20 patients for phase I and 91 for phase II). Like the previous proof-of-concept trial, the radioactive mAb was injected directly into the tumor, and the results for the phase I patients revealed a maximal tolerated dose of 2,590 MBq with serious brain edema for larger doses. For the phase II component, patients received a mean dose of 2,035 MBq with minimal toxicity. Among the patients with glioblastoma, the response rate was 47.2%, and the clinical objective responses were as follows: partial response 12.8%, complete response 1.4%, and no evidence of disease 32.8%. The overall survival median was 19.0 months (Riva et al., 1999).

Yttrium-90, an alternative to iodine-131, has also been trialed with an anti-tenascin BC4 mAb. For 26 patients with recurrent glioblastoma, an amelioration of median survival from 20 months for RIT only to 22 months when RIT was associated with mitoxantrone chemotherapy was demonstrated (Boiardi et al., 2005). These results obtained after an intracranial injection of 185 to 925 MBq of BC-4 mAb was confirmed when RIT was associated with temozolomide chemotherapy (Bartolomei et al., 2004).

**Figure 1:**
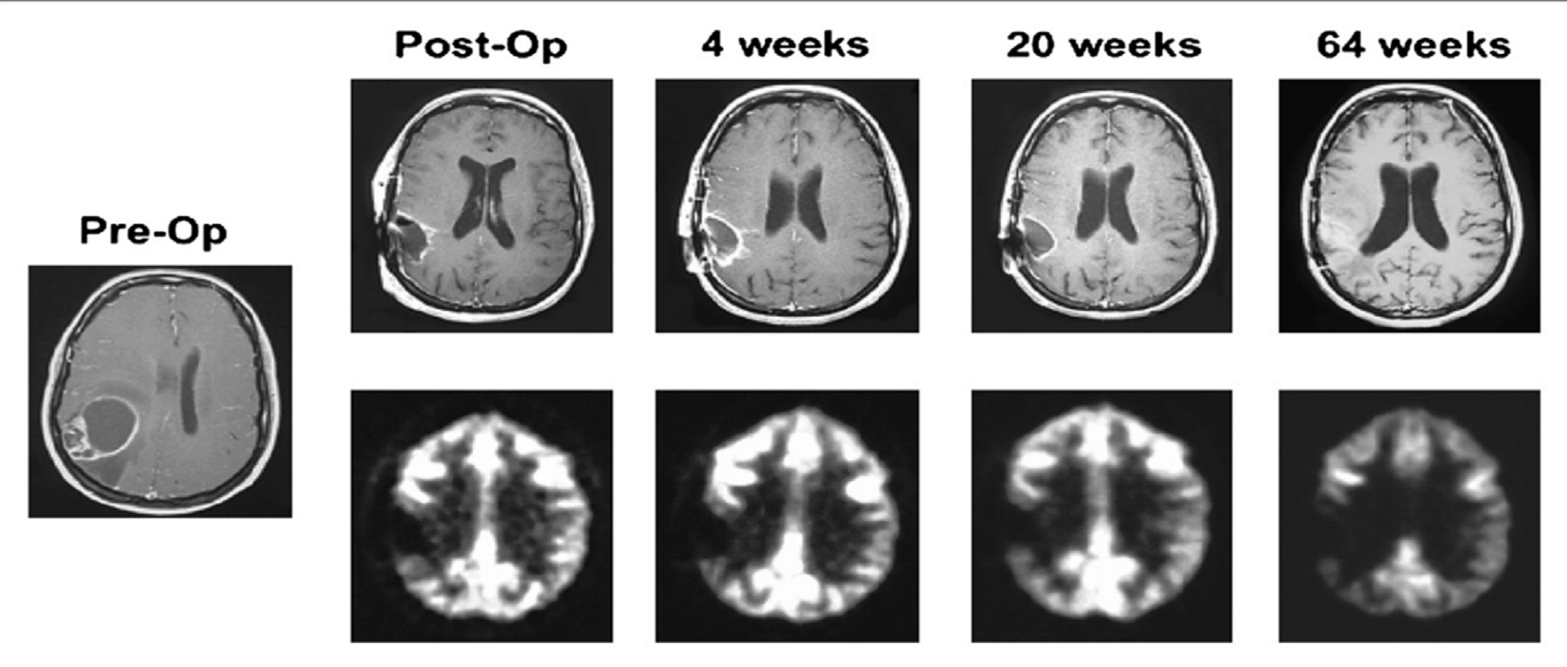
Serial MRI (top and middle) and ^18^F-FDG PET scan results of representative patient after ^131^I-ch81C6 therapy. Corresponding ^18^F-FDG PET scan images (bottom) demonstrate a lack of increased metabolic activity in region of surgically created resection cavity. *This research was originally published in JNM* (Reardon et al., 2006b).

Tenascin targeting appears to be one of the most promising RITs for glioblastoma, and many recent clinical trials using anti-tenascin 81C6 mAb showed similar results to the BC-2 and BC-4 mAbs. The radioisotope mainly used in these clinical trials is the β^−^/γ emitter iodine-131. This RIT protocol consists of an intracranial injection of 370 to 6,660 MBq and increased the median survival to 20.6 months for newly diagnosed glioblastomas and 14.5 months for recurrent disease (**Figure 1**) (Reardon et al., 2006b). This clinical progress was accompanied by reversible grade 3/4 hematologic toxicity and grade 3 neurologic toxicity, which could limit the therapeutic dose in certain cases (Bigner et al., 1995; Akabani et al., 1999; Cokgor et al., 2000; Reardon et al., 2002; Akabani et al., 2005; Reardon et al., 2006a; Reardon et al., 2008).

81C6 mAb is also used with astatine-211, an innovative radioactive alpha emitter. In this clinical trial, which involved 18 patients, 81C6 was radiolabeled with 71–347 MBq of astatine-211 and was injected in the surgically created resection cavity. No patient experienced dose-limiting toxicity (six patients experienced a reversible grade 2 neurotoxicity), and the median survival time for glioblastoma patients was 54 weeks compared with 23–31 weeks observed for patients receiving conventional therapies (Zalutsky et al., 2008).

#### DNA Histone H1 Complex

DNA histone H1 complex is a ubiquitous intracellular antigen exposed in the necrotic core of solid tumors. This targeting is designated as tumor necrosis treatment (TNT) and could be used with a ^131^I iodinated radiolabeled mAb (Shapiro et al., 2006) under the commercial name Cotara^®^. The first-phase I/II RIT clinical trial based on TNT targeting enrolled 51 patients and defined the optimal functional dose for a clinical target volume. The dosing regimen was determined to be 37 to 55.5 MBq/cm^3^ without toxicity (Hdeib and Sloan, 2012). The most frequent adverse events included nervous system disorders such as brain edema (16%), hemiparesis (14%), or headache (14%) (Patel et al., 2005), but a median survival time of 37.9 weeks was reported. In addition, 7 of the 28 recurrent glioblastoma patients survived for more than 1 year.

#### Carbonic Anhydrase

To allow survival within a hypoxic tumor microenvironment, glioblastoma cells overexpress certain enzymes such carbonic anhydrase XII (CA XII) or carbonic anhydrase IX (CA IX) (Mboge et al., 2015; Cetin et al., 2018). CA is a membrane-bound protein overexpressed in glioma but absent from healthy brain tissue and appears to be a potential RIT target. In a preclinical study in mice, a human glioblastoma was xenografted, and the animals were treated by intravenous tail vein injection of a lutetium-177-labeled mAb Fab fragment directed against CA XII. Biodistribution analysis showed a significant (tumor xenograft-specific) accumulation in the brain tumor (3.1% of injected dose 3 h after injection) (Fiedler et al., 2018).

To our knowledge, CA IX is not currently used for therapy in nuclear medicine but was used for molecular imaging of glioblastoma and could be used in the near future for phenotypic imaging and theranostic approach (Li et al., 2015).

#### Cadherin 5

Glioblastoma like many solid tumors is associated with an aggressive and aberrant neovasculature and, in recent years, has become an important target using mAb drugs such as bevacizumab, which targets Vascular endothelial growth factor A (VEGF-A). E4G10 is an mAb targeting vascular endothelial cadherin (e.g., cadherin 5), a molecule specifically expressed at vascular cell–cell junctions in newly forming blood vessels. When labeled with the multiple alpha emitter actinium-225 and intravenously injected into transgenic glioblastoma mice, the E4G10 mAb showed therapeutic efficacy with a significant increase in survival (Behling et al., 2016) and remodeling of the vascular blood–brain-barrier microenvironment, which increased the penetration of chemotherapy drugs like dasatinib (Behling et al., 2016).

#### Integrin α_V_β3

Integrin α_V_β3 is involved in tumor neoangiogenesis and appears to be an oncologic target in various diseases including glioblastoma. Abegrin^®^ is an mAb directed against α_V_β3 and can be used in RIT after radiolabeling with yttrium-90. A proof of concept in a mouse glioblastoma xenograft model showed a partial tumor regression as assessed by image monitoring compared with control groups (Liu et al., 2011).

### Radiopeptide Approach

Peptide receptor radionuclide therapy (PRRT) is an emerging nuclear medicine approach that vectorizes a radionuclide to a specific receptor overexpressed on a tumor using a labeled agonist or antagonist peptide. Various target receptors suitable for PRRT of glioblastoma are being examined in clinical trials with early promising results.

#### Somatostatin Receptors

Somatostatin is a well-known cyclic neuropeptide in nuclear medicine for both diagnosis and therapy of neuro-endocrine tumors. Octreotide derivatives, which mimic somatostatin, present different binding profiles for each somatostatin receptor isotype (SSTR_1–5_). In glioblastoma therapy, a first clinical trial of 43 patients treated with 400 to 3,700 MBq of ^90^Y-DOTA-lanreotide was conducted using a fractionated one-to-six therapy cycle. This proof-of-concept study showed five cases (11.6%) of regressive disease (reduction of more than 25% of the tumor size), 14 cases (32.6%) of stable disease, and 24 patients (55.8%) with progressive disease (increase of more than 25% of tumor size). Furthermore, five patients reported a subjective improvement in quality of life measures (Virgolini et al., 2002). In another trial evaluating the clinical impact of PRRT, an octreotide analog (^90^Y-DOTATOC) was used to treat recurrent high-grade glioma. Three patients with glioblastoma multiform (WHO grade IV) were locally injected *via* a subcutaneous reservoir system implanted into the resection cavity. Patients received 1,660 to 2,200 MBq of ^90^Y-DOTATOC in three or four fractions at an interval of 3 to 4 months. The only adverse effects observed were a reoccurrence of an epileptic seizure for one patient and a mild and transient headache for another. There was a complete remission for one patient and partial remission in the other two patients. The Karnofsky performance score increased by 10% to 40% for the three patients, and they reported an improved quality of life. While two patients died 10 to 13 months after admission in the clinical trial (Heute et al., 2010), the third was alive 4 years after admission.

#### Neurokinin Receptor

Neurokinin type 1 receptors (NK1Rs) appear to be exclusively expressed on the cell surface of gliomas and have been shown to be overexpressed in primary malignant gliomas like glioblastoma (Hennig et al., 1995; Kneifel et al., 2006). The major and physiologic ligand for NK1R is a tachykinin neurotransmitter family member known as substance P.

A pilot study included 20 patients (4 with glioblastoma and 16 with other astrocytoma grade) who received ^90^Y-, ^177^Lu-, or ^213^Bi-radiolabeled substance P into the tumor or into the resected cavity. There was an absence of PRRT drug-related toxicity and 11 months of median survival (range 6–24 months) after therapy initiation. Furthermore, the capacity to modify the radioactive metal in order to optimize tumor growth inhibition and the radionecrotic transformation show promising results for substance P PRRT (Kneifel et al., 2006).

A phase I study whereby 17 patients suffering glioblastoma multiform received repetitive intratumoral injection of ^90^Y-substance P also showed promising results. Fifteen patients stabilized or improved their functional status when PRRT was used as a neoadjuvant therapy with a mean achieved extent of 96% in subsequent resection surgery (due to an improvement of tumor demarcation by radionecrosis) (Cordier et al., 2010). The radiobiologic mechanisms and therapeutic effects of PRRT are directly dependent on the physical properties of the radioactive sources used. As a consequence, it has become potentially appealing to use short-range alpha-particle-emitting radionuclides in glioblastoma PRRT. A pilot alpha PRRT study using ^213^Bi-substance P included five patients and provided a proof of concept for this innovative approach, with no safety concerns and a transformation of primarily non-operable gliomas into resectable gliomas after treatment (Cordier et al., 2010).

**Figure 2:**
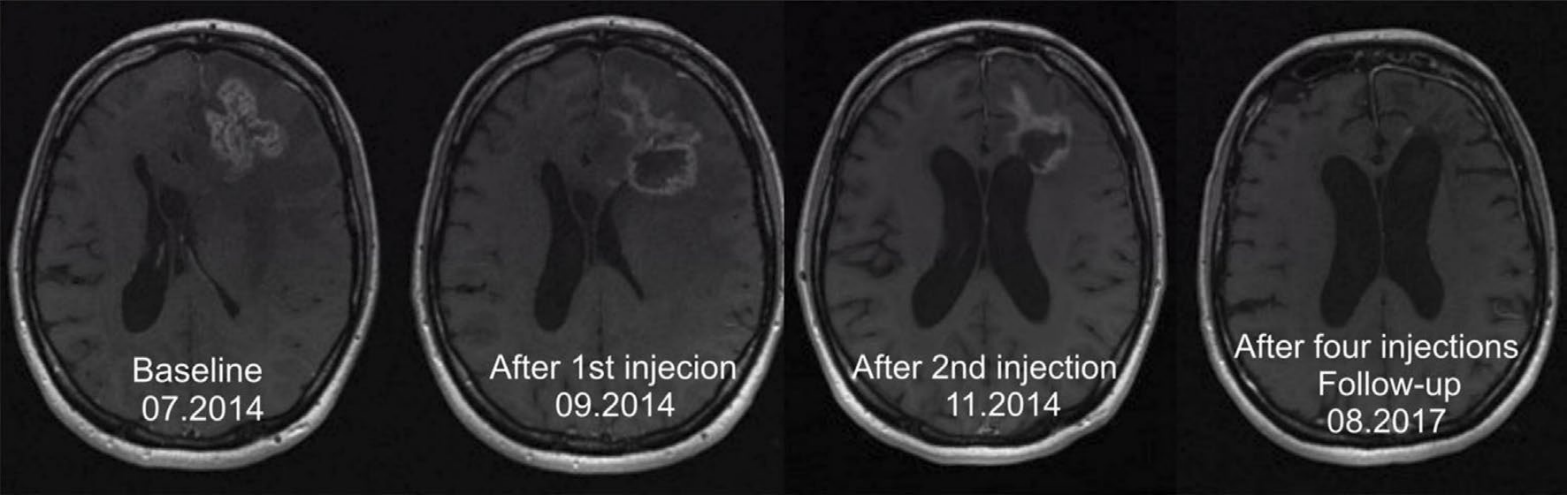
In a 32-year-old woman suffering from an astrocytoma WHO grade II, conversion into a secondary Glioblastoma multiforme (GBM) manifested 10.6 months after initial diagnosis. Following standard treatment consisting of surgery, radiotherapy, and chemotherapy with temozolomide, four cycles of ^213^Bi-DOTA-substance P were applied. The total activity injected amounted to 8.0 GBq of the therapeutic isotope. The T1-weighted enhanced MRI examination revealed shrinkage of the tumor by 32% (Krolicki et al., 2018).

A clinical trial of nine patients suffering recurrent glioblastoma were injected with 1.4 to 9.7 GBq of ^213^Bi-substance P into a resected cavity using a fractionated therapy cycle (one to six). The results supported the pilot study, with a median progression free-survival time of 5.8 months and overall survival time of 16.4 months (**Figure 2**). The median overall survival time from the first diagnostic was 52.3 months, and two of the nine patients (22.2%) are still alive 39 and 51 months after the PRRT initiation (Krolicki et al., 2018). A more recent clinical trial of 20 patients confirms the initial results with only mild and transient adverse reactions, demonstrating that PRRT with ^213^Bi-substance P is safe and well tolerated (Krolicki et al., 2018). The main limitation of ^213^Bi is the short half-life of the isotope (only 45.6 min), which places limits in terms of radiopharmaceutical preparation time, supply chain between preparation and injection, and dosimetric cumulative dose. To resolve this issue, other alpha-emitting isotopes with longer half-lives such as ^225^Ac or ^211^At have been used to label substance P, and preclinical studies are underway (Lyczko et al., 2017; Majkowska-Pilip et al., 2018).

#### Matrix Metalloproteinase

Chlorotoxin is a 36-amino-acid neurotoxin present in the highly toxic venom of the giant yellow Israeli scorpion (*Leiurus quinquestriatus*). It binds to chloride channels (DeBin et al., 1993) and to matrix metalloproteinase-2 with a preference for malignant cells of neuroectodermal origin like gliomas (Cohen-Inbar and Zaaroor, 2016). TM-601 is a recombinant version of chlorotoxin that was radiolabeled with iodine-131 to treat glioblastoma. A phase I single-dose study with 370 MBq of ^131^I-TM-601 administered intracavitarially in 18 high-grade gliomas showed a well-tolerated therapy with no dose-limiting toxicities and promising results (four patients with stable disease at day 180 and two patients without evidence of disease for more than 30 months) (Mamelak et al., 2006).

#### Chemokine Receptor-4

Chemokine receptor-4 (CXCR4) is overexpressed in glioblastoma and is associated with a poor patient outcome (Bian et al., 2007; Tabouret et al., 2015). A first clinical trial of the chemokine receptor ligand Pentixafor^®^ radiolabeled with the positron emitter gallium-68 showed high fixation and provided a nuclear medicine image of glioblastoma (Lapa et al., 2016). An alternative to the diagnostic Pentixafor^®^ is Pentixather^®^, where the radionuclide is a beta-negative emitter (^177^Lu). This radiobiological model is currently in development (Buck et al., 2017) and in the near future could be used for glioblastoma PRRT.

### Radionanoparticle Approach

The use of nanotechnologies in oncology is booming, and glioblastoma nuclear medicine therapy with the use of radionanoparticles appears to be an emerging field. These radioactive nanocarriers can be passive and act as a simple tumor brachytherapy approach or can be active with a specific targeting to vectorize a large amount of radioactivity. In the active case, the targeting is directed against a glioblastoma-specific antigen or receptor as described above for RIT or PRRT.

### Passive Approach

The passive approach of radionanoparticle delivery has been used with different nano-objects such as metallofullerenes, liposomes, or lipid nanocapsules (LNC).

The use of metallofullerene (^177^Lu-DOTA-f-Gd_3_N@C_80_) in an orthotopic xenograft brain tumor model demonstrated its efficacy when the radionanoparticles were delivered by intratumoral convection-enhanced delivery (CED), and it showed an extended survival time of more than 2.5 times that of the untreated group (Shultz et al., 2011).

Liposomes can be loaded with beta-negative emitters such rhenium-186 and provide promising results when administered by CED in an orthotopic glioblastoma rat model (126 days’ median survival compared with 49 days for the control group) (Phillips et al., 2012).

The use of colloidal drugs like LNC loaded with rhenium-188 in a rat orthotopic model induced a remarkable survival benefit (increased median survival time of 257%) after intratumoral stereotactic injection at day 6 and CED injection at day 12 (Vanpouille-Box et al., 2011).

### Active Targeting Approach

A recent approach using radionanoparticles consists of an active targeting approach where the nanoparticles are functionalized and directed against a tumor target. The aim of this active targeting is to optimize the confinement of the radioactivity near to the tumor cells. As an example, LNC can be loaded with rhenium-188 and coupled to a monoclonal antibody directed against the CXCR4 antigen. These CXCR4-recognizing immune-nanoparticles can then irradiate the tumor cells and have been shown to improve the preclinical efficacy in an orthotopic mouse model. Recurrence for the passive protocol was observed at 65 versus 100 days for the active targeting approach, and this appears to be the most effective therapy with the longest measured time to progression (Séhédic et al., 2017).

## Discussion

During the last decade, the knowledge about the phenotypic signature of glioblastoma has increased markedly and resulted in therapeutic progress stemming from improved targeted therapies. Nuclear medicine therapies for glioblastoma have typically used specific vectors to deliver radioactivity to the tumor site. While the initial proof-of-concept studies and human clinical trials have shown encouraging results, some parameters remain to be improved. These include improved efficacy and safety, more localized irradiation of tumor cells, and reduction in bystander irradiation.

These parameters are mostly dependent on the radionuclide and vector choice. Classically, electrons (beta negative and Auger electrons) present an energy-dependent irradiation range. Auger electron emitters (e.g., ^125^I) need an intracellular vectorization close to the tumor cell nucleus to be effective, while beta-negative emitters (e.g., ^131^I, ^90^Y, or ^177^Lu) present a wider irradiation range from several millimeters to centimeters. These physical properties can present advantages, such as large irradiation of tumor margins and “cross fire” effects, which can compensate for the pharmacological heterogeneous distribution of the radiopharmaceutical compound, as well as disadvantages such as incremental local toxicity due to irradiation of healthy tissue. Alpha emitters (e.g., ^213^Bi, ^211^At, or ^225^Ac) deposit a very high energy over a very short range, in the order of 100 µm and can provide a very local irradiation with very low toxicity. The selection of the most appropriate and effective radionuclide in relation to the pathology status (size, dissemination, tumor margin status, etc.) appears to be one of the key factors for successful therapy.

The biodistribution and homogeneous repartition of the vector in the tumor mass are also a requirement for successful treatment. Tumor neoangiogenesis is known to result in morphologically abnormal and highly disorganized structures at the origin of rheologic dysfunction such as arteriovenous shunts or blood flow inversion. As a consequence of this phenomenon, the biodistribution of pharmaceutical compounds (radiopharmaceutical or chemotherapy) is altered inside the tumor, especially for high-molecular-weight drugs like monoclonal antibodies. From a pharmacological point of view, peptide vectorization appears to be more effective in allowing infiltration into the extracellular space of a tumor cell mass. However, clinical studies have shown mAb or high-molecular-weight compounds like radionanoparticles to be effective, and the theoretical “biodistribution barrier” can be bypassed after resection surgery or with co-administration of osmotic drugs like mannitol. For these vectorized approaches (e.g., RIT and PRRT), the key of success is mainly driven on pharmacologic-phenotypic based triad: specific presence of the target, expression level of the target, and pharmacological access to the target. Today, the rising of glioblastoma phenotypic knowledge has permitted to identify various interesting targets such as antigens for RIT and receptors for PRRT. The feasibility of RIT and PRRT in glioblastoma therapy is well established, and the first clinical trial results appear to be promising with well-known targets (e.g., tenascin, EGFR, or neurokinin receptors). Emerging targets such as cadherin, integrin, or chemokine receptors seem to give good results in preclinical and early-phase clinical trials. PRRT is a growing approach, particularly through the easiness where the vector is produced [Good Manufacturing Practice (GMP) compliance automatic synthesis], but presents a risk of direct pharmacological adverse event with the peptide dose used. From a general point of view, it still seems to be early to extrapolate these preliminary but very promising results that need to be clinically confirmed to evaluate the relevance of these approaches and if one (or several) target stands out in terms of therapy benefits.

The surgical administration pathway is also a key factor to optimize in glioblastoma management. If the classical intravenous injection route is used, access to the tumor for the radiotherapeutic drug is *via* the blood–brain barrier; however, other administration pathways are possible. The intratumoral or intra-resected tumoral cavity injection (by port-a-cath system, Rickham, or Ommaya reservoir) is classically used in glioblastoma therapy and shows some benefits in terms of patient outcomes. This intratumoral administration modality can be also modulated in terms of the volume injected, infusion rate, or positive back-pressure. The positive back-pressure used during the injection is known as convection enhanced delivery (CED) and is a promising methodology that ensures an increased interstitial diffusion of the radiopharmaceutical compound around the catheter implantation and a *de facto* increase in the diffusion and homogeneity of the irradiation features (Ung et al., 2015; Vogelbaum and Aghi, 2015). For the unvectorized approaches (e.g., naked nanoparticles in passive approach), the limitation concerns the feasibility of local injection that requires a surgical access. When the neurosurgical procedure allows the injection and if there is no leakage in systemic circulation and a limited diffusion in the extra-cellular matrix, the irradiation could be considered as “classical” brachytherapy. To circumvent the leakage and diffusion risks of passive approach, functionalized nanoparticle (e.g., active approach) presents the advantages of greater confinement of radioactivity inside the tumor or intra-resected tumoral cavity.

One of the limitations of nuclear medicine is the potential for adverse events due to the irradiation of healthy tissues. Increased knowledge of the radiobiological differential mechanisms between healthy and tumoral tissues is associated with recent progress in nuclear medicine therapy and demonstrates the effectiveness of the fractionation approach to increase the tumor irradiation total doses with reduced side effects (Reulen et al., 2015).

## Conclusion

Glioblastoma is the most common primary brain tumor in adults and is typically associated with fatal outcome. Despite recent progress, the survival rate for patients remains poor, and the standard treatment is based on debulking surgery, radiotherapy, and chemotherapy. The current advances in nuclear medicine provide many powerful tools for glioblastoma therapy, and the evolution of biotechnological technologies linked to the molecular knowledge of the pathology and the development of innovative radionuclides has opened the field to new clinical opportunities. Since the first therapeutic injection of a radioactive compound, nuclear medicine applications have been constantly evolving, and new targets like tumoral microenvironment immune checkpoint inhibitors (e.g., CTLA4 or PD1/PDL1 targets) still need to be explored in the field of glioblastoma treatment (Reardon et al., 2016; Huang et al., 2017). In many clinical and preclinical trials, the combination of chemotherapy and nuclear medicine therapy shows an improvement in clinical outcomes by an additive effect of both modalities or by a synergistic effect with a radiosensitization by chemotherapeutic administration (Bartolomei et al., 2004; Milanović et al., 2014).

Many clinical trials demonstrate the efficacy and safety of nuclear medicine approaches, but these have only been assessed in phase I or II clinical trials. These results need to be strengthened, and phase III are trials are necessary to confirm the emerging place of nuclear medicine in the therapeutic arsenal against glioblastoma.

## Author Contributions

All authors listed have made substantial, direct, and intellectual contribution to the work and approved it for publication.

## Funding

This work has been supported by the French National Agency for Research called Investissements d’Avenir *via* grants Labex IRON n°ANR-11-LABX-0018-01 and Equipex Arronax plus n°ANR-11-EQPX-0004.

## Conflict of Interest Statement

The authors declare that the research was conducted in the absence of any commercial or financial relationships that could be construed as a potential conflict of interest.

## References

[B1] Akabani G. Reardon D. A. Coleman R. E. Wong T. Z. Metzler S. D. Bowsher J. E. (2005). Dosimetry and radiographic analysis of 131I-labeled anti-tenascin 81C6 murine monoclonal antibody in newly diagnosed patients with malignant gliomas: a phase II study. J. Nucl. Med. 46 (6), 1042–1051. 15937318

[B2] Akabani G. Reist C. J. Cokgor I. Friedman A. H. Friedman H. S. Coleman R. E. (1999). Dosimetry of 131I-labeled 81C6 monoclonal antibody administered into surgically created resection cavities in patients with malignant brain tumors. J. Nucl. Med. 40 (4), 631–638. 10210222

[B3] Bartolomei M. Mazzetta C. Handkiewicz-Junak D. Bodei L. Rocca P. Grana C. (2004). Combined treatment of glioblastoma patients with locoregional pre-targeted 90Y-biotin radioimmunotherapy and temozolomide. Q. J. Nucl. Med. Mol. Imaging. 48 (3), 220–228. 15499296

[B4] Behling K. Maguire W. F. Di Gialleonardo V. Heeb L. E. M. Hassan I. F. Veach D. R. (2016). Remodeling the vascular microenvironment of glioblastoma with α-particles. J. Nucl. Med. 57 (11), 1771–1777. 10.2967/jnumed.116.173559 27261519 PMC5093034

[B5] Behling K. Maguire W. F. López Puebla J. C. Sprinkle S. R. Ruggiero A. O’Donoghue J. (2016). Vascular targeted radioimmunotherapy for the treatment of glioblastoma. J. Nucl. Med. 57 (10), 1576–1582. 10.2967/jnumed.115.171371 27127217 PMC5050148

[B6] Bian X.-W. Yang S.-X. Chen J.-H. Ping Y.-F. Zhou X.-D. Wang Q.-L. (2007). Preferential expression of chemokine receptor CXCR4 by highly malignant human gliomas and its association with poor patient survival. Neurosurgery 61 (3), 570–578, discussion 578–9. 10.1227/01.NEU.0000290905.53685.A2 17881971

[B7] Bigner D. D. Brown M. Coleman R. E. Friedman A. H. Friedman H. S. McLendon R. E. (1995). Phase I studies of treatment of malignant gliomas and neoplastic meningitis with 131I-radiolabeled monoclonal antibodies anti-tenascin 81C6 and anti-chondroitin proteoglycan sulfate Me1-14 F (ab′)2—a preliminary report. J. Neurooncol. 24 (1), 109–122. 10.1007/BF01052668 8523067

[B8] Boiardi A. Eoli M. Salmaggi A. Lamperti E. Botturi A. Broggi G. (2005). Systemic temozolomide combined with loco-regional mitoxantrone in treating recurrent glioblastoma. J. Neurooncol. 75 (2), 215–220. 10.1007/s11060-005-3030-x 16283445

[B9] Buck A. K. Stolzenburg A. Hänscheid H. Schirbel A. Lückerath K. Schottelius M. (2017). Chemokine receptor—directed imaging and therapy. Methods 130, 63–71. 10.1016/j.ymeth.2017.09.002 28916148

[B10] Casacó A. López G. García I. Rodríguez J. A. Fernández R. Figueredo J. (2008). Phase I single-dose study of intracavitary-administered nimotuzumab labeled with 188Re in adult recurrent high-grade glioma. Cancer Biol. Ther. 7 (3), 333–339. 10.4161/cbt.7.3.5414 18094616

[B11] Cetin B. Gonul I. I. Gumusay O. Bilgetekin I. Algin E. Ozet A. (2018). Carbonic anhydrase IX is a prognostic biomarker in glioblastoma multiforme. Neuropathology 38 (5), 457–462. 10.1111/neup.12485 29952031

[B12] Chen R. Smith-Cohn M. Cohen A. L. Colman H. (2017). Glioma subclassifications and their clinical significance. Neurotherapeutics (US) 14 (2), 284–297. 10.1007/s13311-017-0519-x 28281173 PMC5398991

[B13] Cohen-Inbar O. Zaaroor M. (2016). Glioblastoma multiforme targeted therapy: the chlorotoxin story. J. Clin. Neurosci. 33, 52–58. 10.1016/j.jocn.2016.04.012 27452128

[B14] Cokgor I. Akabani G. Kuan C. T. Friedman H. S. Friedman A. H. Coleman R. E. (2000). Phase I trial results of iodine-131-labeled antitenascin monoclonal antibody 81C6 treatment of patients with newly diagnosed malignant gliomas. J. Clin. Oncol. 18 (22), 3862–3872. 10.1200/JCO.2000.18.22.3862 11078500

[B15] Cordier D. Krolicki L. Morgenstern A. Merlo A. (2016). Targeted radiolabeled compounds in glioma therapy. Semin. Nucl. Med. 46 (3), 243–249. 10.1053/j.semnuclmed.2016.01.009 27067505

[B16] Cordier D. Forrer F. Bruchertseifer F. Morgenstern A. Apostolidis C. Good S. (2010). Targeted alpha-radionuclide therapy of functionally critically located gliomas with 213Bi-DOTA-[Thi8,Met(O2)11]-substance P: a pilot trial. Eur. J. Nucl. Med. Mol. Imaging 37 (7), 1335–1344. 10.1007/s00259-010-1385-5 20157707

[B17] Cordier D. Forrer F. Kneifel S. Sailer M. Mariani L. Mäcke H. (2010). Neoadjuvant targeting of glioblastoma multiforme with radiolabeled DOTAGA-substance P—results from a phase I study. J. Neurooncol. 100 (1), 129–136. 10.1007/s11060-010-0153-5 20217458

[B18] DeBin J. A. Maggio J. E. Strichartz G. R. (1993). Purification and characterization of chlorotoxin, a chloride channel ligand from the venom of the scorpion. Am. J. Physiol. 264 (2 Pt 1), C361–369. 10.1152/ajpcell.1993.264.2.C361 8383429

[B19] Eckel-Passow J. E. Lachance D. H. Molinaro A. M. Walsh K. M. Decker P. A. Sicotte H. (2015). Glioma groups based on 1p/19q, IDH, and TERT promoter mutations in tumors. N. Engl. J. Med. 372 (26), 2499–2508. 10.1056/NEJMoa1407279 26061753 PMC4489704

[B20] Emrich J. G. Brady L. W. Quang T. S. Class R. Miyamoto C. Black P. (2002). Radioiodinated (I-125) monoclonal antibody 425 in the treatment of high grade glioma patients: ten-year synopsis of a novel treatment. Am. J. Clin. Oncol. 25 (6), 541–546. 10.1097/01.COC.0000041009.06780.E5 12477994

[B21] Fiedler L. Kellner M. Gosewisch A. Oos R. Böning G. Lindner S. (2018). Evaluation of 177Lu[Lu]-CHX-A″-DTPA-6A10 Fab as a radioimmunotherapy agent targeting carbonic anhydrase XII. Nuclear Med. Biol. 60, 55–62. 10.1016/j.nucmedbio.2018.02.004 29571067

[B22] Frederick L. Wang X. Y. Eley G. James C. D. (2000). Diversity and frequency of epidermal growth factor receptor mutations in human glioblastomas. Cancer Res. 60 (5), 1383–1387. 10728703

[B23] Hanif F. Muzaffar K. Perveen K. Malhi S. M. Simjee S. U. (2017). Glioblastoma multiforme: a review of its epidemiology and pathogenesis through clinical presentation and treatment. Asian Pac. J. Cancer Prev. 18 (1), 3–9. 10.22034/APJCP.2017.18.1.3 28239999 PMC5563115

[B24] Hdeib A. Sloan A. (2012). Targeted radioimmunotherapy: the role of 131I-chTNT-1/B mAb (Cotara) for treatment of high-grade gliomas. Future Oncol. 8 (6), 659–669. 10.2217/fon.12.58 22764763

[B25] Hennig I. M. Laissue J. A. Horisberger U. Reubi J. C. (1995). Substance-P receptors in human primary neoplasms: tumoral and vascular localization. Int. J. Cancer 61 (6), 786–792. 10.1002/ijc.2910610608 7790112

[B26] Heute D. Kostron H. Guggenberg von E. Ingorokva S. Gabriel M. Dobrozemsky G. (2010). Response of recurrent high-grade glioma to treatment with (90)Y-DOTATOC. J. Nucl. Med. 51 (3), 397–400. 10.2967/jnumed.109.072819 20150267

[B27] Huang J. Liu F. Liu Z. Tang H. Wu H. Gong Q. (2017). Immune checkpoint in glioblastoma: promising and challenging. Front. Pharmacol. 8, 242. 10.3389/fphar.2017.00242 28536525 PMC5422441

[B28] Kaley T. Touat M. Subbiah V. Hollebecque A. Rodon J. Lockhart A. C. (2018). BRAF inhibition in BRAFV600-mutant gliomas: results from the VE-BASKET study. J. Clin. Oncol. 36 (35), 3477–3484. 10.1200/JCO.2018.78.9990 PMC6286161 30351999

[B29] Karsy M. Guan J. Cohen A. L. Jensen R. L. Colman H. (2017). New molecular considerations for glioma: IDH, ATRX, BRAF, TERT, H3 K27M. Curr. Neurol. Neurosci. Rep. 17 (2), 19. 10.1007/s11910-017-0722-5 28271343

[B30] Kneifel S. Cordier D. Good S. Ionescu M. C. S. Ghaffari A. Hofer S. (2006). Local targeting of malignant gliomas by the diffusible peptidic vector 1,4,7,10-tetraazacyclododecane-1-glutaric acid-4,7,10-triacetic acid-substance p. Clin. Cancer Res. 12 (12), 3843–3850. 10.1158/1078-0432.CCR-05-2820 16778112

[B31] Krolicki L. Bruchertseifer F. Kunikowska J. Koziara H. Królicki B. Jakuciński M. (2018). Prolonged survival in secondary glioblastoma following local injection of targeted alpha therapy with 213Bi-substance P analogue. Eur. J. Nucl. Med. Mol. Imaging 45 (9), 1636–1644. 10.1007/s00259-018-4015-2 29713762 PMC6061489

[B32] Krolicki L. Bruchertseifer F. Kunikowska J. Koziara H. Królicki B. Jakuciński M. (2018). Safety and efficacy of targeted alpha therapy with 213Bi-DOTA-substance P in recurrent glioblastoma. Eur. J. Nucl. Med. Mol. Imaging 83, 588. 10.1007/s00259-018-4225-7 30498897

[B33] Lapa C. Lückerath K. Kleinlein I. Monoranu C. M. Linsenmann T. Kessler A. F. (2016). (68)Ga-Pentixafor-PET/CT for imaging of chemokine receptor 4 expression in glioblastoma. Theranostics 6 (3), 428–434. 10.7150/thno.13986 26909116 PMC4737728

[B34] Li J. Zhang G. Wang X. Li X.-F. (2015). Is carbonic anhydrase IX a validated target for molecular imaging of cancer and hypoxia? Future Oncol. (London, UK) 11 (10), 1531–1541. 10.2217/fon.15.11 25963430 PMC4976829

[B35] Li L. Quang T. S. Gracely E. J. Kim J. H. Emrich J. G. Yaeger T. E. (2010). A phase II study of anti-epidermal growth factor receptor radioimmunotherapy in the treatment of glioblastoma multiforme. J. Neurosurg. 113 (2), 192–198. 10.3171/2010.2.JNS091211 20345222

[B36] Li K. Lu D. Guo Y. Wang C. Liu X. Liu Y. (2018). Trends and patterns of incidence of diffuse glioma in adults in the United States, 1973–2014. Cancer Med. 7 (10), 5281–5290. 10.1002/cam4.1757 30175510 PMC6198197

[B37] Liu Z. Wang F. Chen X. (2011). Integrin targeted delivery of radiotherapeutics. Theranostics 1, 201–210. 10.7150/thno/v01p0201 21547160 PMC3086619

[B38] Louis D. N. Perry A. Reifenberger G. Deimling von A. Figarella-Branger D. Cavenee W. K. (2016). The 2016 World Health Organization Classification of Tumors of the Central Nervous System: a summary. Acta Neuropathol. 131 (6), 803–820. 10.1007/s00401-016-1545-1 27157931

[B39] Lyczko M. Pruszynski M. Majkowska-Pilip A. Lyczko K. Was B. Meczynska-Wielgosz S. (2017). (5-11) as potential radiopharmaceutical for glioma treatment. Nuclear Med. Biol. 53, 1–8. 10.1016/j.nucmedbio.2017.05.008 28683361

[B40] Majkowska-Pilip A. Rius M. Bruchertseifer F. Apostolidis C. Weis M. Bonelli M. (2018). In vitro evaluation of 225Ac-DOTA-substance P for targeted alpha therapy of glioblastoma multiforme. Chem. Biol. Drug Des. 92 (1), 1344–1356. 10.1111/cbdd.13199 29611298

[B41] Mamelak A. N. Rosenfeld S. Bucholz R. Raubitschek A. Nabors L. B. Fiveash J. B. (2006). Phase I single-dose study of intracavitary-administered iodine-131-TM-601 in adults with recurrent high-grade glioma. J. Clin. Oncol. 24 (22), 3644–3650. 10.1200/JCO.2005.05.4569 16877732

[B42] Mboge M. Y. McKenna R. Frost S. C. (2015). Advances in anti-cancer drug development targeting carbonic anhydrase IX and XII. Top Anticancer Res. 5 (4), 3–42. 10.2174/9781681083339116050004 30272043 PMC6162069

[B43] Midwood K. S. Chiquet M. Tucker R. P. Orend G. (2016). Tenascin-C at a glance. J. Cell Sci. 129 (23), 4321–4327. 10.1242/jcs.190546 27875272

[B44] Milanović D. Maier P. Schanne D. H. Wenz F. Herskind C. (2014). The influence of retinoic acid and thalidomide on the radio sensitivity of U343 glioblastoma cells. Anticancer Res. 34 (4), 1885–1891. 24692723

[B45] Ostrom Q. T. Liao P. Stetson L. C. Barnholtz-Sloan J. S. (2016). “Epidemiology of glioblastoma and trends in glioblastoma survivorship,” in Glioblastoma. (Philadelphia, USA): Elsevier, 11–19. 10.1016/B978-0-323-47660-7.00002-1

[B46] Patel S. J. Shapiro W. R. Laske D. W. Jensen R. L. Asher A. L. Wessels B. W. (2005). Safety and feasibility of convection-enhanced delivery of Cotara for the treatment of malignant glioma: initial experience in 51 patients. Neurosurgery 56 (6), 1243–1252, discussion 1252–3. 10.1227/01.NEU.0000159649.71890.30 15918940

[B47] Phillips W. T. Goins B. Bao A. Vargas D. Guttierez J. E. Trevino A. (2012). Rhenium-186 liposomes as convection-enhanced nanoparticle brachytherapy for treatment of glioblastoma. Neuro-oncology 14 (4), 416–425. 10.1093/neuonc/nos060 22427110 PMC3309864

[B48] Reardon D. A. Akabani G. Coleman R. E. Friedman A. H. Friedman H. S. Herndon J. E. (2002). Phase II trial of murine (131)I-labeled antitenascin monoclonal antibody 81C6 administered into surgically created resection cavities of patients with newly diagnosed malignant gliomas. J. Clin. Oncol. 20 (5), 1389–1397. 10.1200/JCO.2002.20.5.1389 11870184

[B49] Reardon D. A. Akabani G. Coleman R. E. Friedman A. H. Friedman H. S. Herndon J. E. (2006a). Salvage radioimmunotherapy with murine iodine-131-labeled antitenascin monoclonal antibody 81C6 for patients with recurrent primary and metastatic malignant brain tumors: phase II study results. J. Clin. Oncol. 24 (1), 115–122. 10.1200/JCO.2005.03.4082 16382120

[B50] Reardon D. A. Gokhale P. C. Klein S. R. Ligon K. L. Rodig S. J. Ramkissoon S. H. (2016). Glioblastoma eradication following immune checkpoint blockade in an orthotopic, immunocompetent model. Cancer Immunol. Res. 4 (2), 124–35. 10.1158/2326-6066.CIR-15-0151 26546453

[B51] Reardon D. A. Quinn J. A. Akabani G. Coleman R. E. Friedman A. H. Friedman H. S. (2006b). Novel human IgG2b/murine chimeric antitenascin monoclonal antibody construct radiolabeled with 131I and administered into the surgically created resection cavity of patients with malignant glioma: phase I trial results. J. Nucl. Med. 47 (6), 912–918. 16741299

[B52] Reardon D. A. Zalutsky M. R. Akabani G. Coleman R. E. Friedman A. H. Herndon J. E. (2008). A pilot study: 131I-antitenascin monoclonal antibody 81c6 to deliver a 44-Gy resection cavity boost. Neuro-oncology 10 (2), 182–189. 10.1215/15228517-2007-053 18287339 PMC2613820

[B53] Reulen H.-J. Poepperl G. Goetz C. Gildehaus F. J. Schmidt M. Tatsch K. (2015). Long-term outcome of patients with WHO grade III and IV gliomas treated by fractionated intracavitary radioimmunotherapy. J. Neurosurg. 123 (3), 760–770. 10.3171/2014.12.JNS142168 26140493

[B54] Riva P. Arista A. Sturiale C. Moscatelli G. Tison V. Mariani M. (1992). Treatment of intracranial human glioblastoma by direct intratumoral administration of 131I-labelled anti-tenascin monoclonal antibody BC-2. Int. J. Cancer 51 (1), 7–13. 10.1002/ijc.2910510103 1373410

[B55] Riva P. Arista A. Sturiale C. Tison V. Lazzari S. Franceschi G. (1994). Glioblastoma therapy by direct intralesional administration of I-131 radioiodine labeled antitenascin antibodies. Cell Biophys. 24-25, 37–43. 10.1007/BF02789213 7537631

[B56] Riva P. Franceschi G. Frattarelli M. Riva N. Guiducci G. Cremonini A. M. (1999). 131I radioconjugated antibodies for the locoregional radioimmunotherapy of high-grade malignant glioma—phase I and II study. Acta Oncol. 38 (3), 351–359. 10.1080/028418699431438 10380827

[B57] Séhédic D. Chourpa I. Tétaud C. Griveau A. Loussouarn C. Avril S. (2017). Locoregional confinement and major clinical benefit of 188Re-loaded CXCR4-targeted nanocarriers in an orthotopic human to mouse model of glioblastoma. Theranostics 7 (18), 4517–4536. 10.7150/thno.19403 29158842 PMC5695146

[B58] Shapiro W. R. Carpenter S. P. Roberts K. Shan J. S. (2006). 131I-chTNT-1/B mAb: tumour necrosis therapy for malignant astrocytic glioma. Expert Opin. Biol. Ther. 6 (5), 539–545. 10.1517/14712598.6.5.539 16610983

[B59] Shultz M. D. Wilson J. D. Fuller C. E. Zhang J. Dorn H. C. Fatouros P. P. (2011). Metallofullerene-based nanoplatform for brain tumor brachytherapy and longitudinal imaging in a murine orthotopic xenograft model. Radiology 261 (1), 136–143. 10.1148/radiol.11102569 21813738 PMC3176419

[B60] Stupp R. Mason W. P. van den Bent M. J. Weller M. Fisher B. Taphoorn M. J. B. (2005). Radiotherapy plus concomitant and adjuvant temozolomide for glioblastoma. N. Engl. J. Med. 352 (10), 987–996. 10.1056/NEJMoa043330 15758009

[B61] Tabouret E. Tchoghandjian A. Denicolai E. Delfino C. Metellus P. Graillon T. (2015). Recurrence of glioblastoma after radio-chemotherapy is associated with an angiogenic switch to the CXCL12-CXCR4 pathway. Oncotarget 6 (13), 11664–11675. 10.18632/oncotarget.3256 25860928 PMC4484484

[B62] Tamimi A. F. Juweid M. (2017). “Epidemiology and outcome of glioblastoma,” in Glioblastoma (Brisbane, AU), 143–153. 10.15586/codon.glioblastoma.2017.ch8 29251870

[B63] Thakkar J. P. Dolecek T. A. Horbinski C. Ostrom Q. T. Lightner D. D. Barnholtz-Sloan J. S. (2014). Epidemiologic and molecular prognostic review of glioblastoma. Cancer Epidemiol. Biomarkers Prev. 23 (10), 1985–1996. 10.1158/1055-9965.EPI-14-0275 25053711 PMC4185005

[B64] Ung T. H. Malone H. Canoll P. Bruce J. N. (2015). Convection-enhanced delivery for glioblastoma: targeted delivery of antitumor therapeutics. CNS Oncol. 4 (4), 225–234. 10.2217/cns.15.12 26103989 PMC6088338

[B65] van den Bent M. J. Weller M. Wen P. Y. Kros J. M. Aldape K. Chang S. (2017). A clinical perspective on the 2016 WHO brain tumor classification and routine molecular diagnostics. Neuro-oncology 19 (5), 614–624. 10.1093/neuonc/now277 28339700 PMC5464438

[B66] Vanpouille-Box C. Lacoeuille F. Belloche C. Lepareur N. Lemaire L. LeJeune J.-J. (2011). Tumor eradication in rat glioma and bypass of immunosuppressive barriers using internal radiation with (188)Re-lipid nanocapsules. Biomaterials 32 (28), 6781–6790. 10.1016/j.biomaterials.2011.05.067 21705077

[B67] Virgolini I. Britton K. Buscombe J. Moncayo R. Paganelli G. Riva P. (2002). In- and Y-DOTA-lanreotide: results and implications of the MAURITIUS trial. Semin. Nucl. Med. 32 (2), 148–155. 10.1053/snuc.2002.31565 11965610

[B68] Vogelbaum M. A. Aghi M. K. (2015). Convection-enhanced delivery for the treatment of glioblastoma. Neuro-oncology 17 (suppl 2), ii3–ii8. 10.1093/neuonc/nou354 25746090 PMC4483037

[B69] Zagzag D. Friedlander D. R. Dosik J. Chikramane S. Chan W. Greco M. A. (1996). Tenascin-C expression by angiogenic vessels in human astrocytomas and by human brain endothelial cells in vitro. Cancer Res. 56 (1), 182–189. 8548761

[B70] Zalutsky M. R. Reardon D. A. Akabani G. Coleman R. E. Friedman A. H. Friedman H. S. (2008). Clinical experience with alpha-particle emitting 211At: treatment of recurrent brain tumor patients with 211At-labeled chimeric antitenascin monoclonal antibody 81C6. J. Nucl. Med. 49 (1), 30–38. 10.2967/jnumed.107.046938 18077533 PMC2832604

